# Surgical site infection and its association with rupture of membrane following cesarean section in Africa: a systematic review and meta-analysis of published studies

**DOI:** 10.1186/s40748-020-00122-2

**Published:** 2021-01-02

**Authors:** Alemayehu Gonie Mekonnen, Yohannes Moges Mittiku

**Affiliations:** 1 Department of Nursing, College of Health Science, Debre Berhan University, Po. Box. 445, Debre Berhan, Ethiopia; 2 Department of Midwifery, College of Health Sciences, Debre Berhan University, Po. Box. 445, Debre Berhan, Ethiopia

**Keywords:** Surgical site infection, Rupture of the membrane, Cesarean section, Meta-analysis

## Abstract

**Introduction:**

Surgical site infection occurs within 30 days after a surgical procedure and involves the skin, subcutaneous tissue, and soft tissue. Surgical site infection following cesarean section is a common postoperative complication and is associated with maternal morbidity and mortality in resource-limited settings. Even though the proportion of surgical site infection and some risk factors were reported by kinds of literature, varying results were stated across studies. There is also limited knowledge on the association between postpartum surgical site infection and the rupture of membrane. Hence, this systematic review and meta-analysis was designed to estimate the pooled proportion of surgical site infection and its association with rupture of membrane following cesarean section in Africa.

**Methods:**

Studies published from January 01, 2000 to January 30, 2020 were searched from MEDLINE via PubMed, Scopus, Medscape, Web-science and CINAHL databases to search relevant published articles. We also performed a manual search of reference lists of key articles to retrieve additional relevant articles. Initially, 559 records were identified and 15 studies included in the analysis. The statistical analysis was performed using STATA 11. Heterogeneity between-study was explored by forest plot and inconsistency index (I^2^). The publication bias was checked by a funnel plot and Egger’s test. Pooled estimates of proportion and odds ratio were calculated by a random-effects model with a 95% confidence interval (CI).

**Results:**

The overall pooled proportion of surgical site infection following cesarean section was 10.21% (I^2^ = 86.8, *p* < 0.000; 95% CI = 8.36, 12.06). The odds of developing surgical site infection among women who had the rupture of membrane before delivery were nearly 6 times higher than those who had not a rupture of the membrane (AOR = 5.65, 95% CI: 3.95–8.07).

**Conclusions:**

The proportion of surgical site infections following the cesarean section is relatively high. Women who had rupture of the membrane before delivery were more likely to develop surgical site infections following the cesarean section. Due attention should be given to the provision of prophylactic antibiotics that can reduce surgical site infection after cesarean delivery.

**Supplementary Information:**

The online version contains supplementary material available at 10.1186/s40748-020-00122-2.

## Introduction

Cesarean section (CS) is a surgical procedure where a baby is delivered by cutting through the front wall of the abdomen to open the uterus [1]. CS is often performed in an emergency situation and saves millions of lives worldwide [2, 3]. Globally, the proportion of CS is approximately 15% of all deliveries [4] and the rates are continuing to increase making the burden of surgical site infection an increasingly important issue [5]. As with all surgical procedures, CS can be associated with higher maternal morbidity and mortality during the postpartum period [2, 5].

Surgical site infection (SSI) is defined as an infection that occurs within 30 days after a surgical procedure involving the skin, subcutaneous tissue, and soft tissue [1, 2]. Surgical site infection following CS is a common postoperative complication and is associated with maternal morbidity and mortality specifically in resource-limited settings [6, 7]. It is the foremost predisposing factor for the widespread aversion to the CS in developing countries particularly in Africa [8, 9].

Surgical site infection following CS range from 3 to 15% worldwide [10]. In Africa, the incidence of SSI ranges from 5% in Tunisia [11] to 16.7% in Egypt [12]. The incidence of SSI is usually taken as an indicator of surgical quality [13]. Patients who develop SSIs were five times more likely to be readmitted to hospital and two times more likely to die compared with patients without SSIs [14]. As such, postpartum surgical site infection is a major cause of prolonged hospital stay and poses a burden to the health care system and it also increases an additional costs to mothers and their families [3, 10].

In order to control and prevent post-cesarean wound infection, strict preventative strategies and surgical site infection surveillance systems must be implemented [8]. Previous studies have shown that certain interventions lower SSIs [12]. The world health organization and other studies indicated that quantifying the magnitude of SSIs rate and associated factors can decrease up to 50% of surgical site infections [15, 16]. Furthermore, lower rates of SSIs have been found with preoperative administration of the first-generation antibiotic and by improving the operative room practices [14].

Despite improvements in operating room practices, instrument sterilization methods and better surgical technique, surgical site infections remain a major cause of hospital-acquired infections and rates have been reached their highest levels even in hospitals with most modern facilities [14]. As reported by substantial body of research evidence, patient characteristics and perioperative management were found to be the risk factors for developing SSIs [12–14]. Even though the proportion of surgical site infection and some risk factors were reported by kinds of literature, varying results were stated across studies. There is also limited knowledge on the association between postpartum surgical site infection and the rupture of membrane, especially in developing countries. Therefore, the aim of this systematic review and meta-analysis was designed to estimate the pooled proportion of surgical site infection and its association with rupture of membrane following cesarean section which is currently unknown in Africa.

## Hypothesis/review question

The overarching research question was: what is the best available evidence on the proportion of surgical site infection and its association with rupture of the membrane (ROM) following CS in Africa?

## Methods

This systematic review and meta-analysis is reported in accordance with the meta-analysis of observational studies in epidemiology guidelines [17]. The search strategy focused on studies published from January 01, 2000 to January 30, 2020. The review authors used MEDLINE via PubMed, Scopus, Medscape, Web-science and CINAHL (Cumulative Index to Nursing and Allied Health Literature) databases to search relevant published articles. The search terms were: Infections, Surgical Wound OR Surgical Wound Infections OR Wound Infections, Surgical OR Infection, Surgical Wound OR Surgical Site Infection OR Infection, Surgical Site OR Infections, Surgical Site OR Surgical Site Infections OR Wound Infection, Postoperative OR Wound Infection, Surgical OR Infection, Postoperative Wound OR Infections, Postoperative Wound OR Postoperative Wound Infections OR Wound Infections, Postoperative OR Postoperative Wound Infection) AND (Cesarean section OR Obstetrics case) AND Africa (Additional file 1). These key terms were combined using Boolean operators “AND” and “OR” to narrow the search. A broad search was deliberately conducted to ensure all papers would be retrieved. Besides, the search was supplemented by hand searching of reference lists of key articles to retrieve additional relevant articles. The retrieved articles were exported to Endnote to screen duplicate articles. Then the two review authors assessed and reviewed independently to determine the inclusion articles.

### Study selection criteria

#### Inclusion criteria

For this meta-analysis, the following inclusion criteria were applied:

- Full text cross-sectional, cohort, randomized controlled trial study design
- Studies published after the year 2000 (to minimize the time lag bias)
- Peer-reviewed and published in the English language
- Reported the proportion of SSI and AOR (adjusted odds ratio)
- Studies conducted in Africa

#### Exclusion criteria

The following exclusion criteria were used in screening articles:

- Studies conducted among HIV positive patients as immunocompromised individuals are susceptible to infection
- Studies that repeated findings from the already included studies
- Studies conducted among obese women as those mothers are high risk for SSI

### Study selection and data extraction

Both authors independently ran the search and screened the titles and abstracts against the inclusion/exclusion criteria. Articles satisfying the inclusion criteria were retrieved for full-text evaluation. The authors extracted the data using the pre-determined inclusion criteria. The review authors recorded the data on Microsoft excel and the extracted data included: the authors, year of publication, region, study design, sample size, the proportion of SSI, and adjusted odds ratio of rupture of membrane (Additional file 1). The study selection and data extraction were done from February 01 to March 10, 2020. Data extraction was performed independently by two review authors (AGM and YMM) to ensure accuracy and consistency of included studies (Additional file 2). To be free from other sources of bias, the two investigators assessed the risk of bias and discrepancies were solved through discussion. In addition, the authors communicated the primary authors for clarification (one author replied, but the odds of ROM was not obtained from his data). Both authors assessed the quality of the included studies using the Newcastle-Ottawa Quality Assessment Scale [18].

### Data analysis

The statistical analysis was performed using STATA 11(Stata Corp. USA). Heterogeneity between-study was explored by forest plot (a visual technique that checks whether the confidence intervals of studies overlap with each other) and inconsistency index (I^2^) (a statistical method which describes the percentage of total variation across studies). The I^2^ provides the percentage of variability due to heterogeneity rather than the chance difference or sampling error. Pooled estimates of proportion and odds ratio were calculated by a random-effects model with a 95% CI. The random-effects model which assesses the variability within and between studies was applied to estimate the pooled proportion of surgical site infection and to estimate odds of developing SSI among women who had ROM before delivery. The publication bias was assessed using the funnel plot (which displays effect sizes plotted against the sample size, standard error, conditional variance, or some other measure of the precision of the estimate) and Egger’s test. In the presence of a cloud of data points that is symmetric around the population effect size and has the shape of a funnel, one can conclude as no publication bias [19, 20]. Sensitivity analysis, using the random-effects model, was performed to explore the potential source of heterogeneity (or to identify the studies that affect the overall estimates of the proportion of SSI).

## Results

### Characteristics of the included studies

**Fig. 1:**
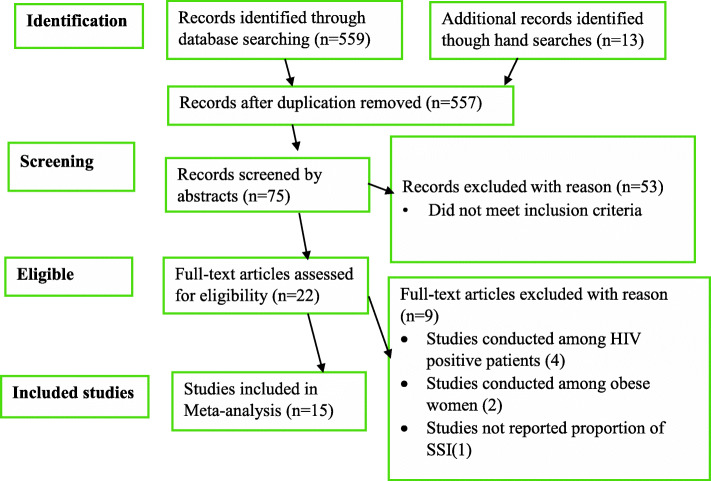
PRISMA flow-diagram that depicts the phases of study selection, March 2020

**Fig. 2:**
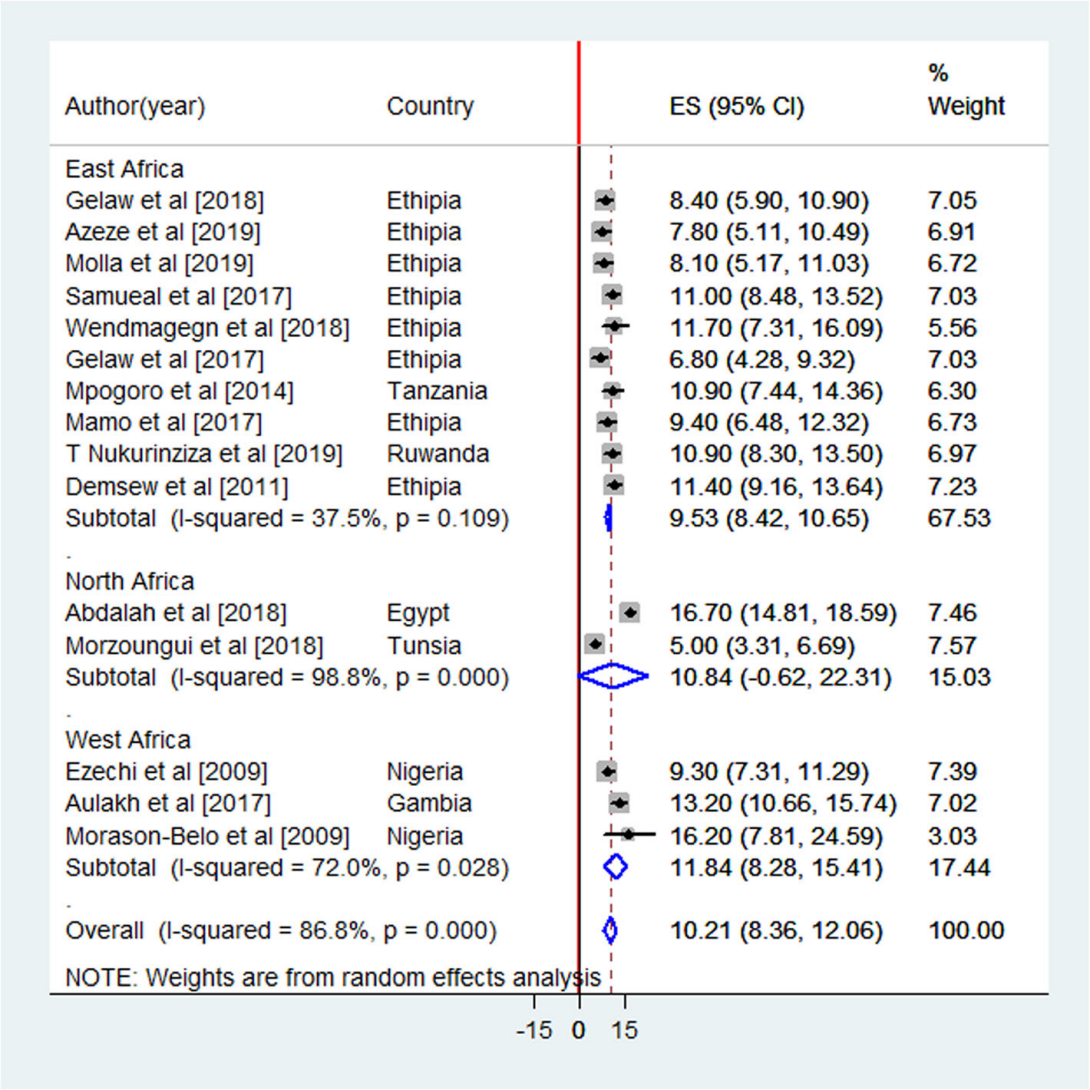
Forest plot of pooled proportion of SSI following cesarean section from the random effects model, March 2020

Initially, 559studies were retrieved, of which 64 were identified as being potentially eligible for this review and analysis. Finally, 15 studies (Fig. 1) comprising of 8098 women who underwent a CS were included in the pooled analysis of the proportion of SSI following CS (Fig. 2). Of 15 included articles, 11 studies were cross-sectional, 3 studies were cohort, and one article was a randomized control trial. The studies included women who gave birth through CS with a sample size ranging from 74 [21] to 1500 [12]. Among the articles included in this analysis, the least reported SSI proportion was 5% (11) and the highest proportion was 16.7% [12]. Most (10) of the studies were conducted in East Africa [4, 8, 9, 13, 22–27], two were from North Africa [11, 12] and three studies were from West Africa countries [5, 21, 28]. The quality of the studies included in this meta-analysis was accurately evaluated by using the Newcastle-Ottawa quality scale (one of the most standardized quality assessment tool used worldwide) [29–32]. All studies achieved a score of at least five stars, indicating good study quality of included studies.

### Heterogeneity and publication bias

**Fig. 3:**
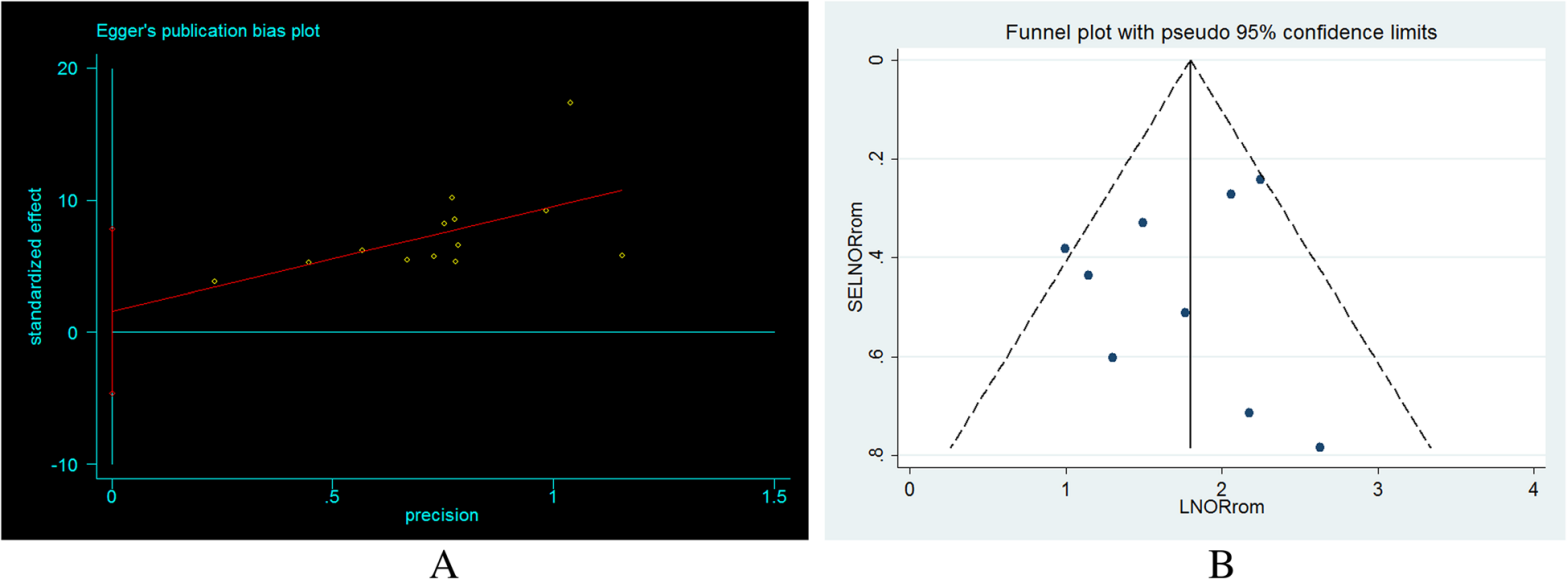
Egger’s publication bias plot (**a**), funnel plot showing publication bias (inverted symmetrical funnel plot) (**b**) among studies, March 2020

The included studies were assessed for heterogeneity and publication bias. Accordingly, the heterogeneity test showed a substantial heterogeneity among studies and the true variability among the 15 studies other than chance was 88.5% (I^2^ = 88.5%). To resolve the presence of heterogeneity, subgroup analysis, sensitivity analysis and random-effect model were applied (Fig. 2). The publication bias was checked by a funnel plot and Egger’s test, and the plot has a symmetric inverted funnel shape showing no evidence of variability in effect sizes from studies and publication bias (Fig. 3). Egger’s test also provides no evidence for small-study effects and publication bias among studies (*p* = 0.581) (Table 1) (Fig. 3).

**Table 1:**
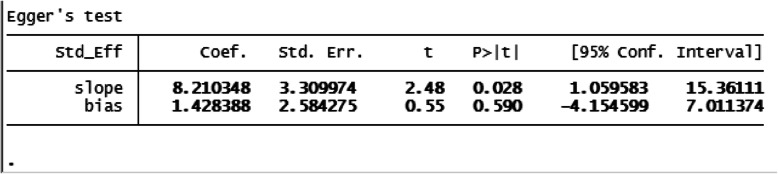
Egger’s test for small-study effects and publication bias among studies, March 2020

### Sensitivity analysis

**Fig. 4:**
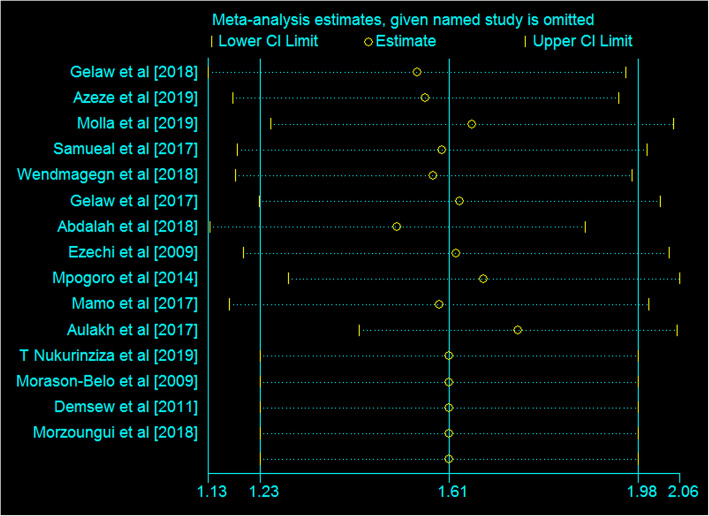
A sensitivity analysis of the effect of one study on the pooled estimates, March 2020

A sensitivity analysis of the 15 studies was also conducted to test whether a particular study was responsible for the presence of high heterogeneity. The output showed that the estimated points of the sensitivity analysis were within the confidence interval for the pooled estimate of the meta-analysis that shows no statistical source of heterogeneity among the included studies (Fig. 4).

### The pooled proportion of surgical site infection

The pooled proportion of surgical site infection is presented in a forest plot (Fig. 1). The proportion of SSI following CS in each study ranged from 5.0% (95% CI = 3.31, 6.69) [11] to 16.7% (95% CI = 14.81, 18.59) [12]. In this meta-analysis, the overall pooled proportion of surgical site infection following CS was 10.21% (I^2^ = 86.8, *p* < 0.000; 95% CI = 8.36, 12.06). In the subgroup analysis by region, the estimated proportion of SSI following CS was 9.5% (I^2^ = 37.5%, *p* < 0.109; 95% CI = 7.42, 10.65), 10.8% (I^2^ = 98.8, *p* < 0.000; 95% CI = − 0.62, 22.31) and 11.8% (I^2^ = 72.8, *p* < 0.028; 95% CI = 8.28, 15.41) in East, North, and West African studies, respectively (Fig. 2).

### The association between rupture of membrane and SSI following CS

Nine studies were included to estimate odds of developing SSI among women who had ROM before delivery [4, 8, 12, 13, 22–24, 27, 28]. The characteristics of the included studies are described above. The adjusted odds ratio of the articles ranged from 2.70 (95% CI = − 0.62, 22.31) [4] to 13.90 (95% CI = − 0.62, 22.31) [8]. The included studies were assessed for heterogeneity and publication bias. The analysis showed low heterogeneity among studies (I^2^ = 42.7%). The publication bias was checked by a funnel plot and Egger’s test, and funnel shape showed no evidence of variability in effect sizes from studies (Fig. 3). Egger’s test also showed no evidence of publication bias among studies (*p* = 0.514) (Table 2).

**Table 2:**
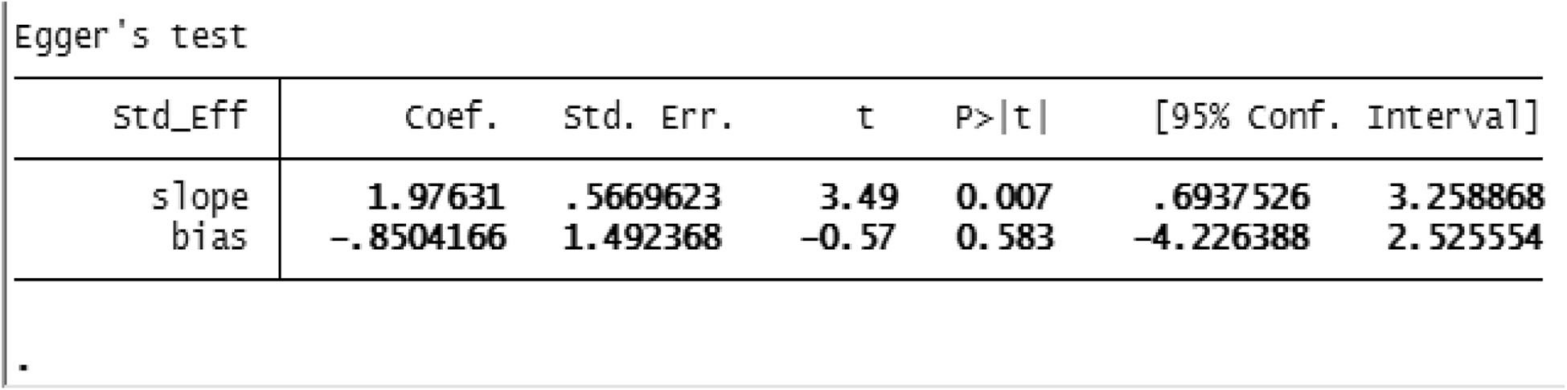
Egger’s test for small-study effects and publication bias among studies, March 2020

**Fig. 5:**
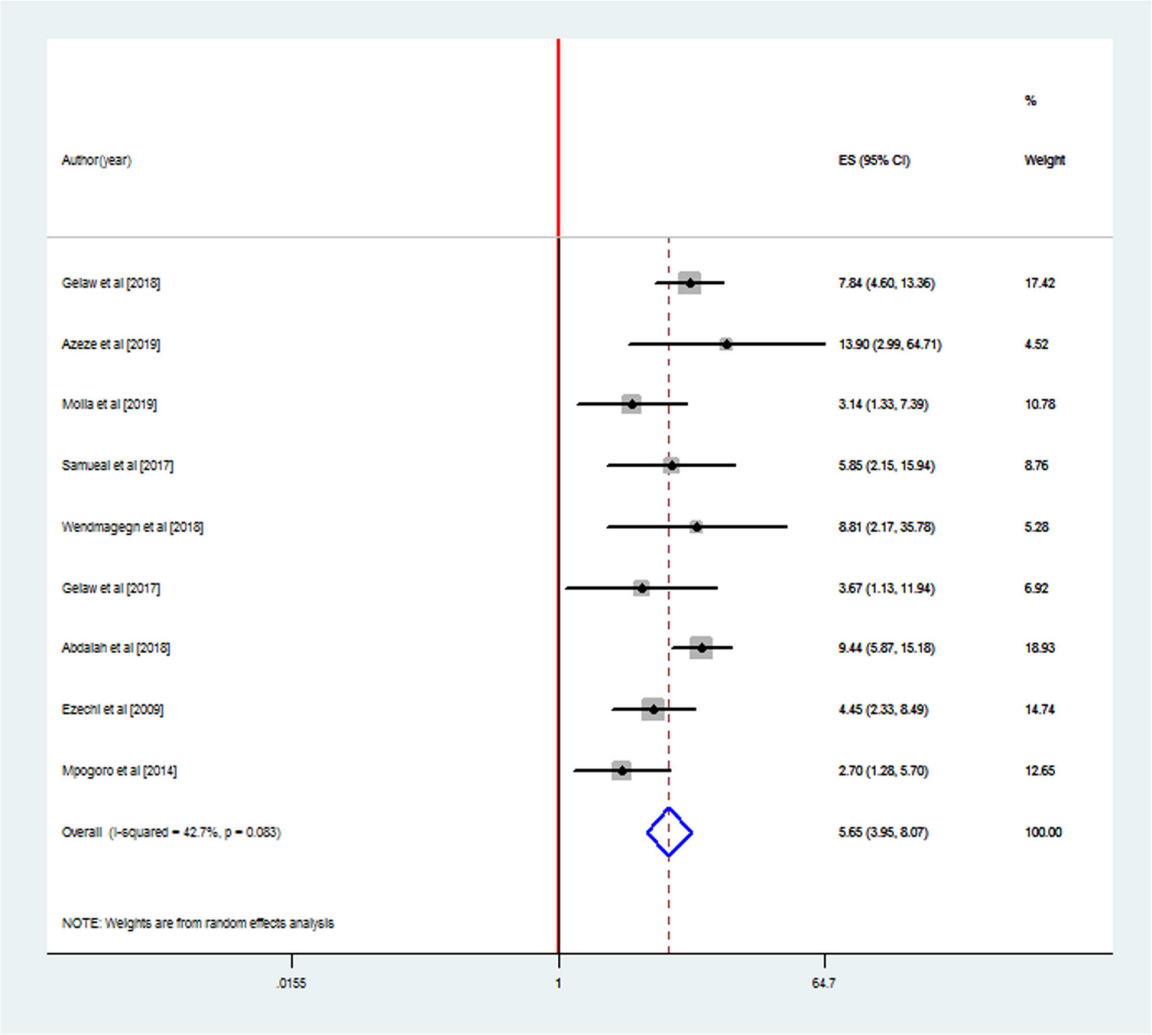
Forest plot of pooled estimates of an association between ROM and SSI, March 2020

The pooled analysis of these 9 studies showed that the presence of an association between ROM and SSI following CS. The odds of developing SSI among women who had ROM before delivery were nearly six times higher than those who had not ROM (AOR = 5.65, 95% CI: 3.95–8.07) (Fig. 5).

## Discussion

This systematic review and meta-analysis, to the best of our information, is the first meta-analysis conducted in Africa to determine the pooled proportion of SSI following CS and its association with the rapture of membrane during late pregnancy. Accordingly, the overall pooled proportion of surgical site infection following CS was 10.21%. Also, the subgroup analysis of this meta-analysis revealed that the proportion of surgical site infection following CS slightly varied across the regions of the included study area. The proportion of SSI following CS was higher in West Africa regions (11.84%) than in East Africa regions (9.53%). This pooled proportion of SSI following CS was higher than the study reported in Sub-Saharan Africa (7.3%) [3]. A systematic review of surgical site infection by Lakhan et al. reported that 1.9 to 11.2% of SSI among women operated for cesarean delivery [33]. A study from El Minya general hospital, Egypt reported a relatively higher proportion of SSI following CS (16.7%) [12]. This high incidence of SSI following CS could be due to a continuing rise in cesarean birth rate and unsafety of perioperative surgical procedures in the health facilities during cesarean-delivery. As clearly reported in research findings, when the CS rate increases, the women at risk of developing SSI will also increase [34, 35]. Complicated pregnancies might be at higher risk for SSI following CS [35]. Lack of perioperative antibiotics and basic infrastructure might also contribute high incidence of surgical site infection following a cesarean section [35].

In this pooled meta-analysis, nine of 15 studies were included in the analysis to estimate odds of developing SSI following CS among women who had rupture of membranes before cesarean delivery. Accordingly, the odds of developing SSI among women who had ROM before cesarean delivery were nearly six times higher than those who did not have the rupture of membranes. Even though they were not reviews, this finding was supported by other studies [12, 26, 33]. This could be reasoned out that women who had ROM before cesarean delivery were more exposed to SSI. However, there had also been many conflicting studies about ROM and developing SSI following CS and the conclusiveness of this evidence might be compromised by these non-significant findings. A study published in 2006 found that there was no significant association between ruptured membranes before surgery and developing SSI [36]. This uniformity of the findings might arise from the inherent limitations of fragmented published articles. Even though we have pooled adjusted estimates of the association between the rupture of membrane and SSI following CS, this meta-analysis might have some limitations. The included studies were restricted in reports published in English and relevant articles available in other languages may be missed. This analysis was based only on published studies and important data might be missed from unpublished studies.

## Conclusions

The proportion of surgical site infections following CS is relatively high. Women who had rupture of the membrane before delivery were more likely to develop surgical site infections following CS. Due attention should be given to the provision of prophylactic antibiotics that can reduce surgical site infection after cesarean delivery.

## Supplementary Information

**Additional file 1.** MEDLINE via PubMed, Scopus database search for surgical site infection following cesarean section, March 2020.

**Additional file 2.** Summary of retrieved studies included in the analysis, March 2020.

**Additional file 3.** PRISMA checklist that ensure consistency and uniformity in reporting of systematic review, March 2020.

## Data Availability

The datasets used and/or analyzed during the current study are available from the corresponding author on reasonable request.

## Abbreviations

AOR: Adjusted Odds Ratio
CI: Confidence Interval
CS: Cesarean Section
I^2^: Inconsistency Index
HIV: Human Immunovirus
ROM: Rupture of Membrane
SSI: Surgical Site Infection
