# Supplementary material for: Surgical site infection and its association with rupture of membrane following cesarean section in Africa: a systematic review and meta-analysis of published studies
Source: Matern Health Neonatol Perinatol. 2021 Jan 2;7:2. doi: 10.1186/s40748-020-00122-2 (PMC7777267; doi:10.1186/s40748-020-00122-2)
Supplement: Supplementary file 1 — Additional file 1. MEDLINE via PubMed, Scopus database search for surgical site infection following cesarean section, March 2020. [file 40748_2020_122_MOESM1_ESM.docx]

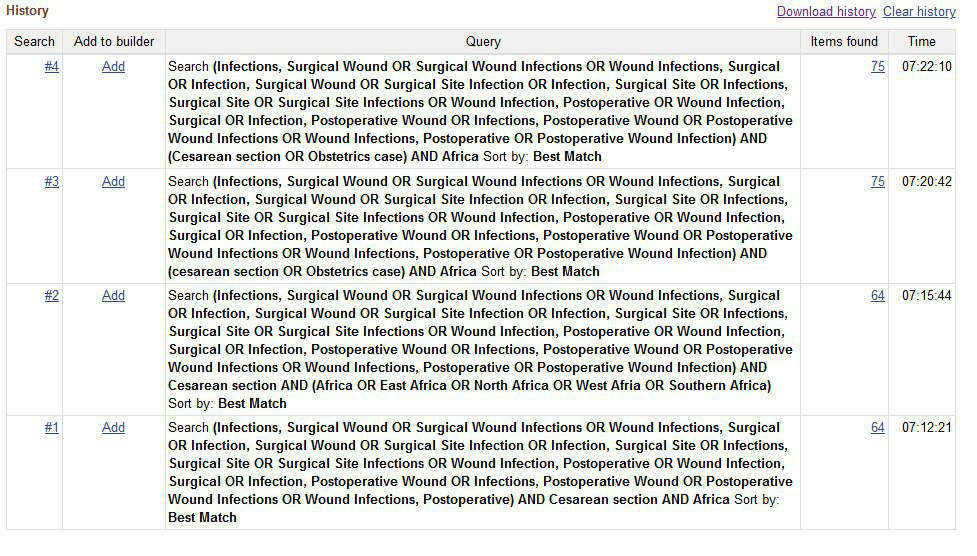


Scopus review


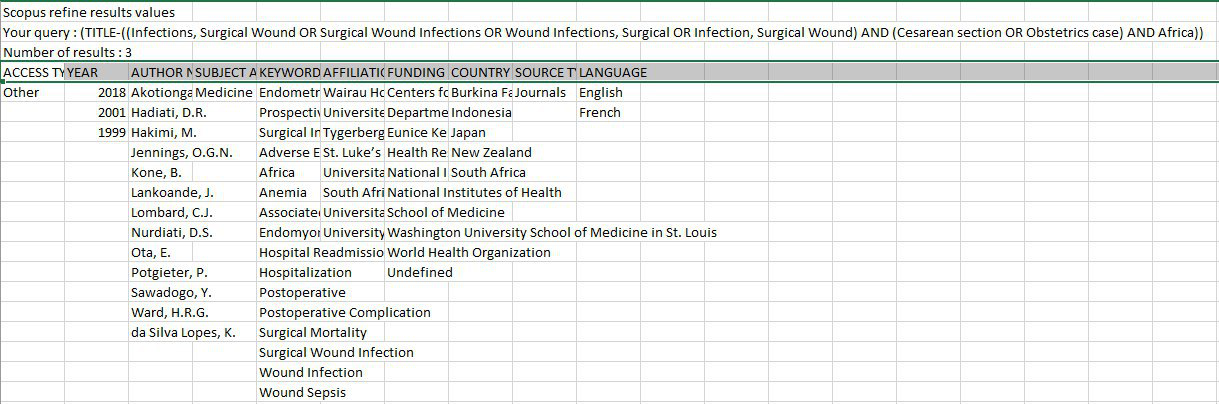


Additional file 1. MEDLINE via PubMed, Scopus database search for surgical site infection following cesarean section, March 2020.
