# Supplementary figures and images for: Surgical site infection and its association with rupture of membrane following cesarean section in Africa: a systematic review and meta-analysis of published studies
Source: Matern Health Neonatol Perinatol. 2021 Jan 2;7:2. doi: 10.1186/s40748-020-00122-2 (PMC7777267; doi:10.1186/s40748-020-00122-2)

Additional file 2. Summary of retrieved studies included in the analysis, March 2020.


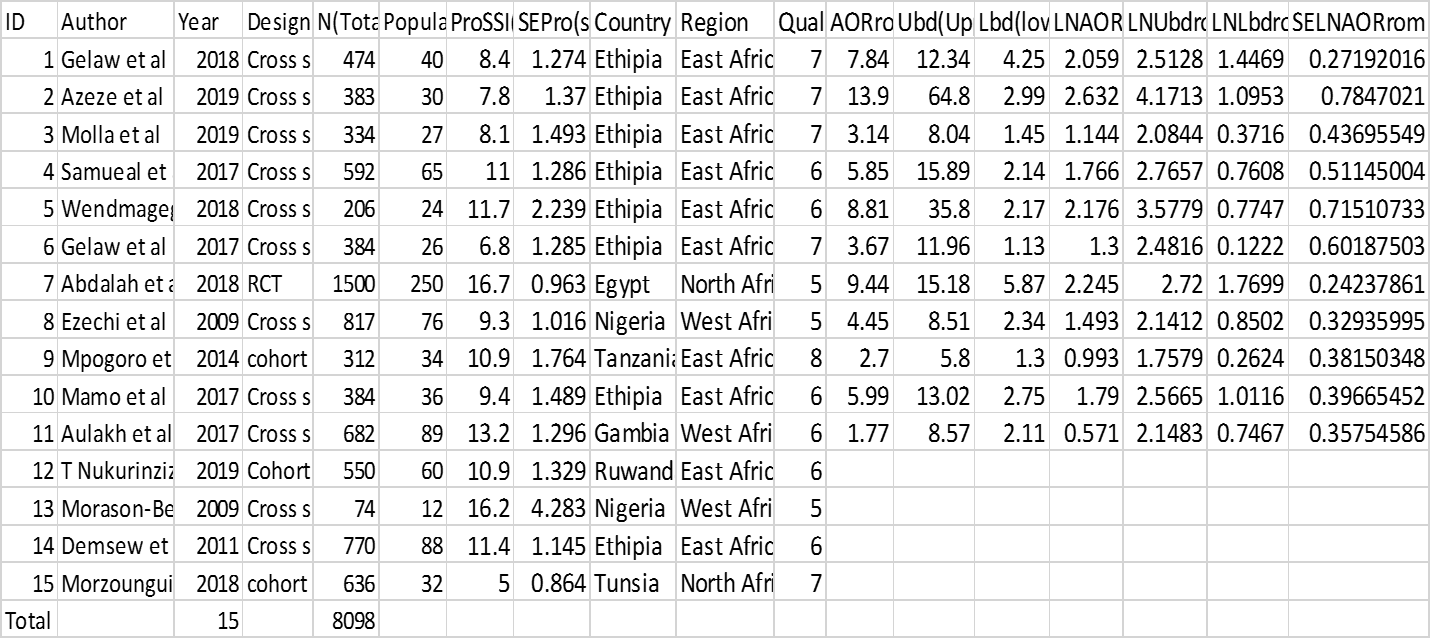

Supplement: Supplementary file 2 — Additional file 2. Summary of retrieved studies included in the analysis, March 2020. [file 40748_2020_122_MOESM2_ESM.docx]
